# Efficacy of the meningococcal vaccine against *Neisseria gonorrhoeae*: a randomised clinical trial (MenGO)

**DOI:** 10.1038/s41541-026-01422-y

**Published:** 2026-04-22

**Authors:** Caroline Thng, Sharareh Eskandari, Fengyi Jin, Ian Hughes, Andrew E. Grulich, Maree O’Sullivan, Evgeny A. Semchenko, Kate L. Seib

**Affiliations:** 1 Gold Coast Sexual Health, Gold Coast Hospital and Health Service, Southport, QLD Australia; 2 Institute for Biomedicine and Glycomics, Griffith University, Southport, QLD Australia; 3 The Kirby Institute, University of New South Wales, Sydney, NSW Australia; 4 Gold Coast University Hospital, Southport, QLD Australia

**Keywords:** Diseases, Immunology, Medical research, Microbiology

## Abstract

*Neisseria gonorrhoeae* (NG) causes significant morbidity globally, and no licensed vaccine currently exists. Observational studies suggest the four-component meningococcal B (4CMenB) vaccine may provide cross-protection against NG. We conducted MenGO, an open-label, randomized controlled trial to evaluate the efficacy, safety, and immunogenicity of 4CMenB against NG in gay and bisexual men (GBM). Participants were randomized 1:1 to receive two doses of 4CMenB, or no vaccine, and were followed for 2 years with 3-monthly visits. 4CMenB vaccination did not significantly reduce gonorrhoea incidence (incidence rate ratio 0.78 (95% confidence interval (CI) 0.40-1.51;*p* = 0.457), and no study-related serious adverse events occurred. The vaccine was immunogenic against NG, with higher serum bactericidal activity (hSBA) median titres at 6 months in vaccinated compared to unvaccinated participants (4 [interquartile range (IQR) 2-8] vs 2 [IQR 2-2];*p* = 0.0021). However, hSBA titres did not increase in participants with an NG infection at the time of vaccination (2 [IQR 2-2] to 2 [IQR 2-4];*p* = 0·999), whereas those without NG infection showed an increase from baseline to 6 months (2 [IQR 2-4] to 6 [IQR 2-16];*p* = 0.0089). This study is limited by its small sample size based on an assumed vaccine efficacy of >67% and was impacted by the COVID-19 pandemic. These findings provide important information for current 4CMenB vaccines programmes and gonorrhoea vaccine development and implementation.

## Introduction

*Neisseria gonorrhoeae* (NG) is a major public health concern worldwide, with a substantial burden on sexual and reproductive health. In 2020, more than 82 million people were diagnosed with gonorrhoea and case numbers continue to rise, with growing concerns of extensively antibiotic-resistant strains^1^. Gonorrhoea disproportionately affects gay and bisexual men (GBM), people in low-income countries, racial and ethnic minorities, and Indigenous populations.

In Australia, gonorrhoea notifications increased by 155% between 2014 and 2023, with 69% of infections in males. Gonorrhoea incidence in GBM is high, with a reported incidence of 36.4 per 100 person‑years among GBM attending sexual health clinics^2^ and 37.8 per 100 person years in GBM taking PrEP^3^. Risk factors for acquisition of gonorrhoea in GBM include a history of NG infection^4^, high number of sexual partners, using injecting drugs, living with HIV infection and being <30 years of age^5^. If left untreated, NG infection can lead to severe sequelae that include pelvic inflammatory disease, chronic pelvic pain, adverse reproductive health outcomes (ectopic pregnancy and tubal infertility) in women, epididymitis in men, and disseminated gonococcal infection in both women and men^6^. NG is also associated with an increased risk of acquiring HIV infection^7^. The ongoing emergence of increasingly multidrug-resistant NG (MDR-NG) raises concerns for access and availability of effective treatments, resulting in increased healthcare costs, and potential for increased risk of complications and poor long-term outcomes^1^.

The Global Health Strategies on HIV, Hepatitis, and sexually transmitted infections (STIs) have set targets to reduce global NG incidence by 20% in the next 2 years, and 90% by 2030, with a focus on innovative approaches for the prevention of infections, in particular the development of vaccines^1^. Despite over a century of research, only four gonococcal vaccine candidates have been tested in human clinical trials, and none have been shown to protect against gonorrhoea^8,9^. However, observational studies suggest that outer membrane vesicle (OMV) meningococcal B vaccines may provide cross-protection against NG^10^.

4CMenB (Bexsero®) is a four-component, OMV and recombinant protein-based vaccine indicated for protection against group B *Neisseria meningitidis* since 2013. Over the past decade, several observational studies have consistently reported between 33-47% protection by 4CMenB against gonorrhoea^11–14^, with a recent systematic review and meta-analysis estimating vaccine effectiveness of around 33–34% after a 2-dose vaccine schedule^10^. Data from South Australia, where 4CMenB has been routinely offered to adolescents since 2019, indicated 44.3% protection against gonorrhoea using chlamydia patients as controls during the first 4 years of the programme, with vaccine efficacy lower in those >48 months post vaccination (26.2%) compared to those vaccinated within ≤48 months (46·0%)^15^. The only published randomised controlled trial (RCT) evaluating the efficacy of 4CMenB in GBM at high risk of gonorrhoea (the ANRS174 DOXYVAC trial), reported an adjusted hazard ratio of 0.78 (95% confidence interval (CI) 0.60–1.01; *p* = 0.061) against the first episode of NG infection^16^.

The mechanism of 4CMenB cross-protection against NG is attributed to the close genetic relatedness of NG and *N. meningitidis*, with several 4CMenB antigens being surface-exposed proteins on *N. meningitidis* that have homologues in NG. 4CMenB induces antibodies in humans that recognize gonococcal proteins^17^, and data from a female mouse model of NG genital tract infection demonstrated that 4CMenB vaccination accelerated clearance significantly and reduced the NG bacterial burden following vaginal challenge^18^.

Based on available data from these observational studies, vaccination programmes using 4CMenB as a preventative strategy against gonorrhoea have been implemented in Galicia, Spain in adults aged 18-65 years at risk of infection^19^ and in England for individuals at greatest risk of infection, including GBM^20^. Our open-label, randomised controlled trial aimed to assess the efficacy, safety, and immunogenicity of 4CMenB against NG in GBM with high-risk of infection.

## Methods

### Study design and participants

The MenGO study protocol has been published previously^21^. MenGO was a single-centre, open-label RCT conducted at the Gold Coast Sexual Health Service, Australia. Eligible participants were GBM between the ages of 18 and 50 years and included those taking HIV pre-exposure prophylaxis (PrEP) or with a confirmed NG infection in the previous 3 months, to reflect an at-risk population that may be prioritised for vaccine implementation programmes. Key exclusion criteria were chosen for patient safety^22^ or where they may affect study endpoints, and included: any contraindications for vaccination, prior 4CMenB vaccination or severe immunocompromise. Participants were followed up 3-monthly for 2 years, and participants who discontinued, withdrew, or were lost to follow-up were not replaced.

An independent data safety and monitoring board provided trial oversight. The trial was approved by the Gold Coast Health Human Research Ethics Committee and is registered with the Australian and New Zealand Clinical Trials Registry (ACTRN12619001478101). All participants provided written informed consent prior to any study procedures.

### Randomisation and intervention

Participants were randomly assigned 1:1 to receive 4CMenB (vaccinated arm) or no treatment (unvaccinated arm) at the baseline visit. Block randomisation was performed using the *ralloc* command in Stata 18 using random blocks of size 2 to 10. A statistician who was not involved in study procedures provided the randomization, which was provided to study investigators by sequentially numbered, opaque sealed envelopes. Participants and study investigators were unblinded to study allocations. The use of standardised laboratory testing conducted at a central facility reduced the likelihood of substantial bias in primary outcomes.

Participants allocated to the vaccine arm received 2 doses of 0.5 mL 4CMenB intramuscularly at baseline and month 3 visit, with a minimum interval of 28 days between doses and no maximum interval. This 2-dose schedule is in accordance with Australian Therapeutic Goods Administration (TGA) registration and has a documented safety profile. Concurrent administration of other vaccines except for COVID-19 vaccines was allowed. All other vaccines administered during the study period were recorded.

### Procedures

Eligible participants were identified by study-site clinicians and referred to the study team for information about the study and to provide informed consent. Scheduled study visits were conducted every 3 months over 2 years. Additional unscheduled visits were undertaken to manage any side effects after vaccination, missed study visits, if there were any technical issues with samples or for any clinical care outside of the scheduled visits.

At all visits, pharyngeal, rectal, and urine samples were collected unless a test had already been performed within the last 14 days with verifiable results. Samples were tested for NG and *Chlamydia trachomatis* by nucleic acid amplification tests (NAAT) using the Cobas 6800 CT/NG assays at Pathology Queensland. NG-positive NAAT tests (cycle threshold (Ct) of ≤40) were confirmed with a Pathology Queensland in-house GeneXpert NG assay testing for NG2 and NG4 targets. Laboratory-confirmed NG infections diagnosed elsewhere were also included. NG cultures were performed according to local clinic procedures if there was urethral discharge, a confirmed NG diagnosis (prior to treatment) or sexual contact with a person with confirmed NG infection. At all study visits, participants with a positive NG test were recalled to the clinic for treatment as per standard of care. Participants with urethral symptoms or who were contacts of a confirmed NG case at a study visit were treated on the day of the visit. All other clinically indicated tests and procedures were allowed, including but not limited to clinical examination, microscopy, other relevant tests, and treatment based on clinical presentation (e.g., testing for other sexually transmitted infections (STI) and blood-borne viruses (BBV)). Blood samples were collected at baseline (0), 3, 6, 12, and 24 months for immunological analysis. Other data collected at each study visit included all concurrent medications, any antibiotic use at the study visit or within the last 14 days, and symptoms of NG infection. Following the publication of the DOXYVAC study^16^, the potential impact of prophylactic use of doxycycline on NG was also investigated. No doxycycline was prescribed for gonorrhoea prophylaxis (doxyPEP) throughout the study, and was only used for the treatment of urethritis, epidemiological treatment as a contact of chlamydia or when chlamydia infection was confirmed. The number of sexual partners and condom use since the past visit were also collected to evaluate potential behavioural risk compensation due to the open-label study design. Participants were provided with adverse event reporting diaries and screened for adverse events (AEs) at every study visit to document any safety events throughout the study period, including any solicited and unsolicited local and systemic AEs associated with vaccination.

### Outcomes

The primary outcome was the incidence of all-episode NG infections diagnosed by NAAT during the study follow-up, allowing for multiple episodes of infection per participant. The study follow-up period started from 30 days after the second 4CMenB dose (vaccinated arm) or 30 days after the second scheduled visit (unvaccinated arm) and ended on the date of the last study visit, scheduled or not, where an NG test was performed. Multi-site infections were counted as a single episode if diagnosed at the same study visit.

Secondary outcomes were safety and immunogenicity of 4CMenB. The safety and tolerability of 4CMenB were measured by the number of serious adverse events (SAEs) reported, where the cause was determined to be related to 4CMenB administration. All grading of AEs followed the Common Terminology Criteria for Adverse Events (CTCAE) Version 5·0^23^.

4CMenB-induced immune responses against NG were measured by serum bactericidal activity assays using human complement (hSBA) as described previously^24^. Briefly, heat-inactivated sera from study participants were serially diluted and incubated with NG strain 1291 and exogenous human complement for 60 min at 37 °C. Assays were plated on GC agar and incubated overnight to enable bacterial enumeration.

### Statistical analysis

Our study was the first RCT to commence recruitment to investigate 4CMenB against gonorrhoea, and with the paucity of any other RCTs registered at the time, our study was designed to inform the feasibility and design of future efficacy trials as well as exploratory immunology endpoints. Our sample size was calculated to detect a 67% reduction in NG incidence. This vaccine efficacy is higher than that reported in observational studies (33-47%)^11–14^ but aligns with the greater than 50% vaccine efficacy outlined in the WHO preferred product characteristic to achieve a significant effect in reducing gonococcal infection prevalence at a population level^25^. Based on an estimated NG incidence among GBM taking PrEP at the study site of 32.1 infections/100 person years (PY) in 2018-2019, with 80% power at a significance level of 0.05 based on the proportions test (G*Power 3·1). To account for 5% loss to follow-up consistent with other RCTs at the study site, 130 participants were recruited.

Statistical analyses were performed using Stata version 18·5 (StataCorp, College Station, TX, USA) and Prism version 10.4.1 (GraphPad). All missing data were treated as missing, and there was no missing data imputation used for analysis.

For the primary outcome of all-episode NG, NG incidence was calculated per-protocol as the total number of episodes diagnosed during study follow-up divided by total person-years (PY) accrued and presented as the number of episodes per 100 PY, with the exact 95% confidence interval (CI). Participants were considered lost to follow up and excluded from analysis if they did not attend at least one visit during the follow-up period. Participants who received only one vaccine dose (if allocated to the vaccinated arm), died or withdrew from the study were also excluded from analysis. NG incidence was compared between the two study arms overall, and by anatomical site, culture positivity, presence of symptoms, NAAT cycle threshold, and concurrent chlamydia infection. As there is evidence to support a protective effect with a single vaccine dose^26^, a intention to treat analysis (ITT) was carried out, including all participants in the vaccinated arm who received at least one dose of vaccine and attended at least one follow-up visit and all participants in the placebo arm who attended at least one visit after baseline. In both per-protocol and ITT analyses, the Andersen–Gill approach was used to allow multiple failures in the same participant to be counted during study follow-up and robust standard errors were calculated. First episode analysis of NG infections where study follow up ended on the date of the first NG positive test, or the date of the last study visit in those who were not diagnosed with NG was also performed to enable comparison with another similar RCT, DOXYVAC^16^.

Due to the open-label nature of the study design, potential confounders associated with NG risk were assessed, including time-fixed baseline (e.g. demographics, HIV status, and previous STIs) and time-varying covariates (e.g. number of sexual partners, condom use, incident chlamydia diagnosis, and doxycycline use). This was to ensure that any differences in the treatment arms post randomisation, which may independently affect infection risk regardless of vaccination, were considered. Univariable and multivariable Poisson regression models were developed to assess risk factors associated with incident NG infection in addition to assignment of study arms, which was entered as a priori. The effect of vaccination was measured as the incidence rate ratio (IRR), which is essentially the rate of infections in the vaccinated group divided by the rate in the unvaccinated group. The IRR was estimated using Poisson regression with a log link and offset by the log of the total risk time per participant. All covariates with a *p*-value of 0.10 or less in univariable analysis were considered for inclusion in the multivariable model, alongside vaccination status. Potential interactions between selected covariates and study arm were also explored in relation to incident NG infection. The unadjusted IRR and adjusted IRR (aIRR) of the association between 4CMenB and incident gonorrhoea with 95% CI per-protocol were reported.

Differences in adverse event outcomes were analysed using chi-squared tests. Bactericidal titres are reported as the reciprocal of the lowest dilution of participant serum that induced ≥50% killing relative to the no sera control. The lower limit of detection is 4, and hSBA titres <4 were reported as 2. Kruskal-Wallis tests and one-way ANOVA were used to compare titres with 95% CI between groups.

## Results

### Study recruitment and follow up

**Fig. 1:**
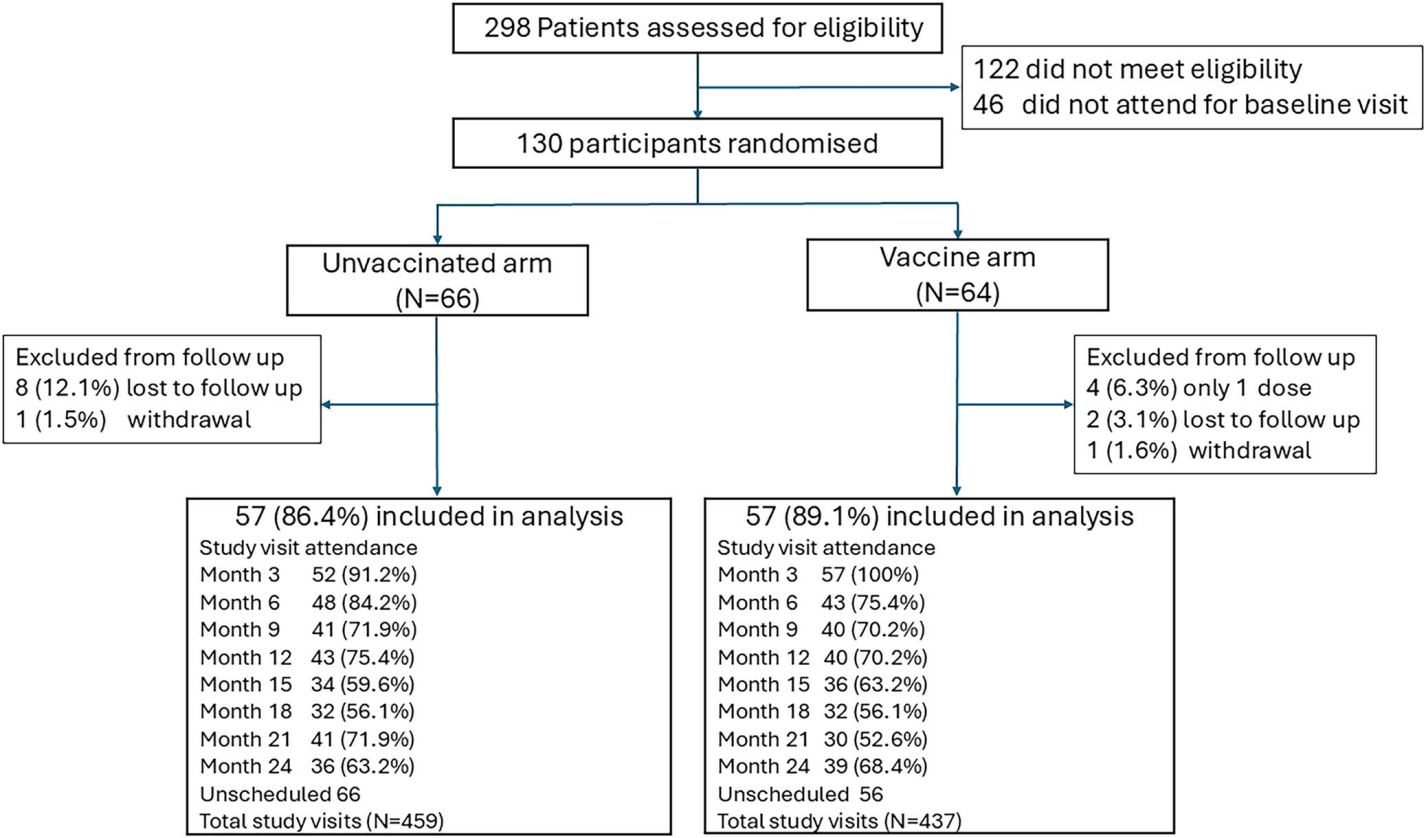
Trial profile–per-protocol analysis. Follow up was defined as all visits from 30 days after the second dose of 4CMenB was given (vaccine arm) or 30 days after the date of second scheduled study visit (unvaccinated arm). Percentages in follow up describe the proportion of participants attending each study visit. Participants were excluded from analysis if they did not attend for any visits during the follow up period, withdrew consent or only attended for 1 dose of vaccine (if allocated to vaccine arm).

From January 21, 2020, to April 29, 2022, 298 GBM were assessed for eligibility, and 130 participants were recruited, including 66 participants randomised to receive no vaccine and 64 participants randomised to receive 4CMenB (Fig. 1).

For per-protocol analysis, 16 participants were excluded as they were lost to follow up (LTFU) (*n* = 10), withdrew (*n* = 2) or only received a single dose of vaccine (*n* = 4) (Fig. 1). For primary outcome analyses, 57/66 (86.4%) participants in the unvaccinated arm were included with 459 follow up visits (77.4 PY) compared to 57/64 (89.1%) in the vaccine arm with 437 follow up visits (81.1 PY).

In comparison, in the ITT analysis (Supplementary Fig. 1), all participants who were randomised to the vaccine arm and received at least one dose of vaccine were included, and only participants who did not attend at least one follow up visit were excluded. 7 participants were excluded from analyses (2 withdrawal and 5 did not attend for further visits after baseline (LTFU), and hence 61 unvaccinated participants were included in the analysis with 465 study visits (101.2 PY) and 62 participants in the vaccine arm with 454 study visits (104.9 PY) (Supplementary Fig. 1). The per-protocol and ITT analyses showed similar results in vaccine efficacy and there were no differences in terms of age, HIV status, and history of gonorrhoea in those who were included and not included in the ITT analyses.

### Baseline participant characteristics

The baseline demographics and clinical characteristics were balanced between the two study groups, with no significant differences between groups (Table 1). The median age of participants was 33.4 years (interquartile range (IQR) 27–41). All participants were cis-gender men, 97 (85.1%) were White Caucasian, 100 (91.0%) HIV-negative participants were taking PrEP. The median number of casual partners in the 3 months prior to starting the study was 2 (IQR 1–5), and all but 3 participants had had sex within the last 3 months. Condom use was low, with 77 (67.5%) participants reporting less than 50% condom use. There were 90 (78.9%) participants with a history of a bacterial STI, 74 (64.9%) with previous NG infection, and 32 (28.1%) with NG infection in the previous 3 months. There were 4 (3.5%) participants living with HIV infection (PLHIV), all of whom had CD4 counts >200 and were taking antiretroviral therapy. At baseline, 17 participants (14.9%) had NG, 8 (7.0%) had chlamydia, and 4 (3.5%) had syphilis infection. One participant with NG at baseline received treatment at the same time as vaccination.

**Table 1:** Baseline participant demographics in the per-protocol analysis population

	Unvaccinated arm (*N* = 57)		Vaccinated arm (*N* = 57)	
	*n*	%	*n*	%
Age				
18–29	21	36.8	17	29.8
30–34	11	19.3	18	31.6
≥35	25	43.9	22	38.6
Ethnicity				
White	49	86.0	48	84.2
Other	8	14.0	9	15.8
HIV status				
Negative	55	96.5	55	96.5
Positive	2	3.5	2	3.5
Number of recent casual partners (last 3 months)				
less than 5	52	91.2	42	73.7
6–10	3	5.3	8	14.0
11 or more	0	0.0	7	12.3
Condom use				
<50%	39	68.4	38	66.7
≥50%	18	31.6	19	33.3
*N. gonorrhoeae* (last 3 months)				
No	45	78.9	37	64.9
Yes	12	21.1	20	35.1
History of STI				
Gonorrhoea	35	61.4	39	68.4
Chlamydia	34	59.6	32	56.1
Genital herpes	9	15.8	6	10.6
Syphilis	12	21.1	18	31.6
Hepatitis C	0	0.0	0	0.0
STI at Baseline				
Gonorrhoea	8	14.0	9	15.8
Chlamydia	3	5.3	5	8.8
Syphilis	2	3.5	2	3.5
PrEP use (HIV-negative only)				
No	4	7.3	6	10.9
Yes	51	92.7	49	89.1

*STI* sexually transmitted infections, *PrEP* HIV pre-exposure prophylaxis.

### NG infection following vaccination

There were 80 (70.2%) participants with NG infection at any visit during the total study period, including baseline, of whom 22/80 (27.5%) had recurrent NG infections, 18/80 (22.5%) had multi-site NG infections, and 19/80 (23.8%) had concurrent NG and chlamydia infections. In the per-protocol analysis, NG incidence was 34.9 per 100 PY (27 episodes in 18 of 57 participants) in the unvaccinated and 27.1 per 100 PY (22 episodes in 13 of 57 participants) in the vaccinated arm (IRR 0.78 [95% CI 0.40–1.51]; *p* = 0.457) (Table 2). In the ITT analysis, NG incidence was 30.6 per 100 PY (31 episodes in 19 of 61 participants) in the unvaccinated arm and 26.7 per 100 PY (28 episodes in 18 of 62 participants) in the vaccine arm (IRR 0.87 [95% CI 0.48–1.58]; *p* = 0.652) (Table 2).

**Table 2:** Primary efficacy outcomes (per-protocol and intention to treat (ITT)) and secondary efficacy outcomes (per-protocol analysis only)

	Incident NG	Person-years	Incidence Per 100 PY	Incidence rate ratio	95% CI	*P* value
NG episodes (per-Protocol)						0.457
Unvaccinated	27	77.4	34.8	1	—	
Vaccinated	22	81.1	27.1	0.78	0.40–1.51	
NG episodes (ITT)						0.652
Unvaccinated	31	101.2	30.6	1	—	
Vaccinated	28	104.9	26.7	0.87	0.48–1.58	
By anatomic site						
Urethra						0.454
Unvaccinated	5	77.4	6.5	1	—	
Vaccinated	9	81.1	11.1	1.72	0.42–7.08	
Anorectum						0.326
Unvaccinated	16	77.4	20.7	1	—	
Vaccinated	11	81.1	13.6	0.66	0.28–1.52	
Oropharynx						0.642
Unvaccinated	12	77.4	15.5	1	—	
Vaccinated	10	81.1	12.3	0.80	0.30–2.09	
Culture positive						0.906
Unvaccinated	13	77.4	16.8	1	—	
Vaccinated	13	81.1	16.0	1.05	0.15–2.45	
Symptoms						
*Symptomatic*						0.804
Unvaccinated	11	77.4	14.2	1	—	
Vaccinated	13	81.1	16.0	1.13	0.43–2.93	
*Asymptomatic*						0.193
Unvaccinated	16	77.4	20.7	1	—	
Vaccinated	9	81.1	11.1	0.54	0.21–1.37	
Concurrent Chlamydia						0.348
Unvaccinated	7	77.4	9.0	1	—	
Vaccinated	4	81.1	4.9	0.54	0.11–2.15	

Cumulative incidence of gonorrhoea from 30 days after the second 4CMenB dose (vaccinated arm) or 30 days after the second scheduled visit (unvaccinated arm) ending on the date of the last study visit, scheduled or not, where an NG test was performed. Multi-site infections were counted as a single episode if diagnosed at the same study visit.

No significant difference was observed between the unvaccinated and vaccinated arms for NG incidence by anatomical site (urogenital IRR 1.72 [95% CI 0.42–7.08]; *p* = 0.454, anorectal IRR 0.66 [95% CI 0.28-1.52]; *p* = 0.326, oropharyngeal IRR 0.80 [95% CI 0.30–2.09]; *p* = 0.642), the presence of symptoms (symptomatic IRR 1.13 [95% CI 0.43–2.93]; *p* = 0.804), NG culture positivity (IRR 1.05 [95% CI 0.15–2.45]; *p* = 0.906), mean NAAT cycle threshold (Ct) (28.1 [standard deviation (SD) 4.0] vs 29.4 [SD 4.0]; *p* = 0.238), or NG cases with concurrent chlamydia infection (IRR 0.54 [95% CI 0.11–2.15]; *p* = 0.348) (Table 2).

**Fig. 2:**
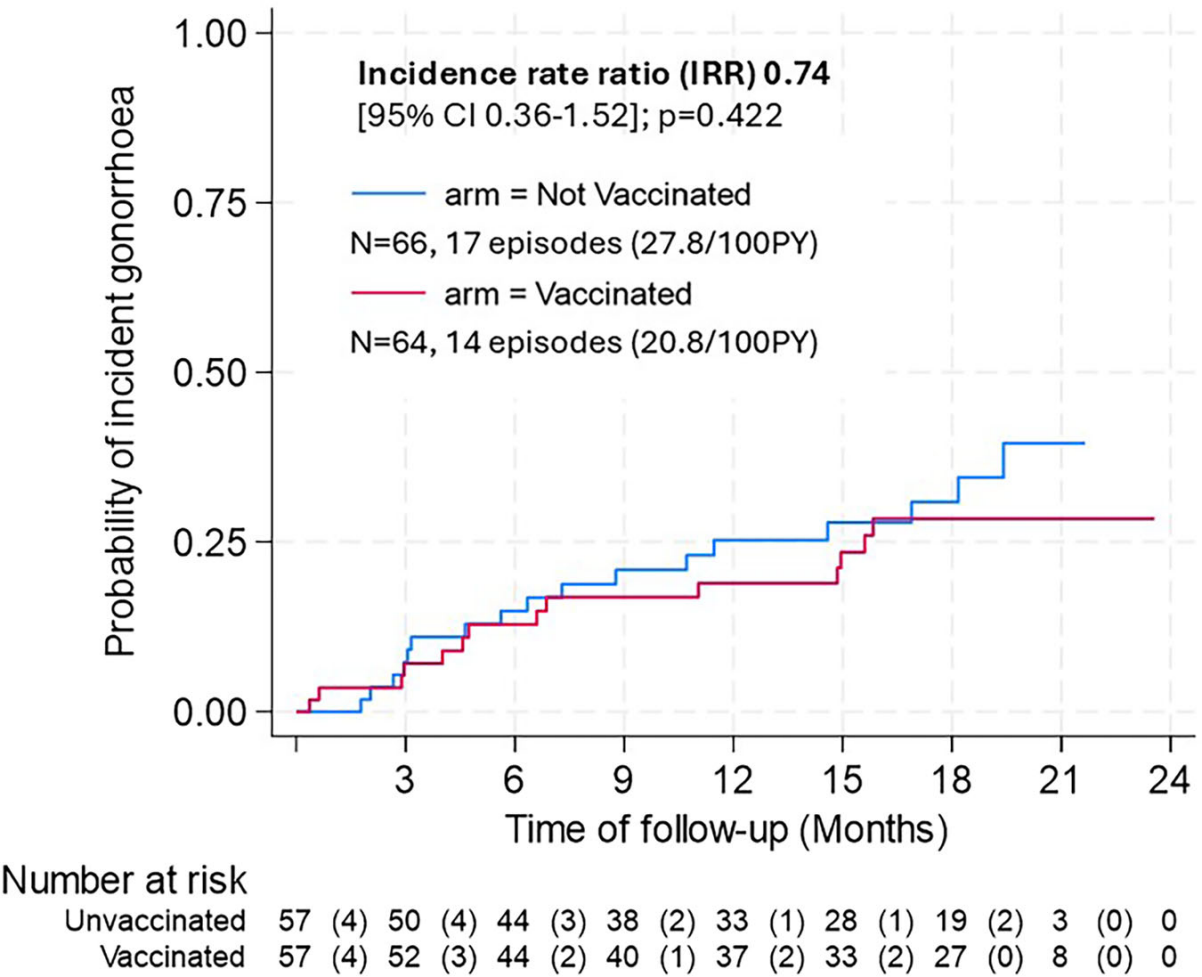
Kaplan-Meier failure estimates for first episode infection. First episode analysis: study follow up ended on the date of the first NG positive test, or the date of the last study visit in those who were not diagnosed with gonorrhoea.

When only first episode infections were considered (where data were censored upon first NG infection), NG incidence was 27.8 per 100 PY in the unvaccinated group (17 episodes) compared to 20.7 per 100 PY (14 episodes) in the vaccinated group (IRR = 0.74 [95% CI 0.36–1.52]; *p* = 0.422) (Fig. 2).

### Risk factors and confounders of NG incidence

Risk factors associated with NG incidence included taking PrEP and also having gonorrhoea in the 3 months prior to enrolment (IRR = 2.59 [95% CI 1.24–5.40]; *p* = 0.016), a previous history of gonorrhoea (IRR = 1.83 [95% CI 0.90–3.69]; *p* = 0.093), high number of casual partners in the previous 3 months (IRR = 2.58 for ≥11 casual partners [95% CI 1.29–5.17]; *p* = 0.004), chlamydia infection (IRR = 1.79 [95% CI 0.92–3.48]; *p* = 0.085) and doxycycline use (IRR = 1.85 [95% CI 1.03–3.32]; *p* = 0.038) (Table 3).

**Table 3:** Univariable analysis of risk factors associated with incident NG infection (all episodes)

	Person-years	*n*	Incidence (per 100 person-years)	Incidence rate ratio (IRR)	95% CI	*P* value
Age						0.754
18–29	44.1	13	29.5	1	—	
30–34	37.5	15	40.0	1.36	0.55–3.32	
35 and above	76.9	21	27.3	0.93	0.40–2.17	
HIV status						
Negative	154.3	49	31.8	—	—	
Positive	4.3	0	0.0	—	—	
Inclusion criteria (baseline risk factors for NG infection)						0.016
Taking PrEP	117.1	29	24.8	1	—	
Gonorrhoea^b^	19.0	6	31.6	1.28	0.49–3.34	
Both	21.8	14	64.2	2.59	1.24–5.40	
History of gonorrhoea						0.093
No	54.8	11	20.1	1	—	
Yes	103.7	38	36.6	1.83	0.90–3.69	
History of chlamydia						0.774
No	71.7	21	29.3	1	—	
Yes	86.8	28	32.2	1.10	0.57–2.12	
History of syphilis						0.790
No	116.5	37	31.8	1	—	
Yes	42.1	12	28.5	0.90	0.41–1.99	
History of herpes						0.108
No	139.9	39	27.9	1	—	
Yes	18.6	10	53.8	1.93	0.87–4.31	
History of hepatitis C						—
No	158.5	49	30.9	—	—	
Yes	0	0	—	—	—	
Number of regular partners (last 3 months)						0.356
0	44.1	14	31.7	1	—	
1	50.4	11	21.8	0.69	0.32–1.47	
2-5	49.4	20	40.5	1.28	0.60–2.72	
6 or more	3.5	2	57.8	1.82	0.62–5.35	
Number of casual partners (last 3 months)						0.004
0-5	108.6	27	24.9	1	—	
6-10	22.3	10	44.8	1.80	0.94–3.44	
11 or more	17.1	11	64.2	2.58	1.29–5.17	
Condom use						0.908
<50%	46.34	14	30.2	1	—	
≥50%	112.0	35	31.3	1.03	0.58–1.84	
Chlamydia diagnosis						0.085
No	137.6	39	28.3	1	—	
Yes	19.7	10	50.8	1.79	0.92–3.48	
Doxycycline dispensed or prescribed during study^a^						0.038
No	137.1	38	27.7	1	—	
Yes	21.4	11	51.4	1.85	1.03–3.32	

^a^No doxycycline was used as gonorrhoea prophylaxis (doxyPEP) throughout the study, and was only used for the treatment of urethritis, epidemiological treatment as a contact of chlamydia or where chlamydia infection was confirmed.

^b^Gonorrhoea diagnosis in the 3 months prior to enrolment.

In the multivariable analysis, the adjusted IRR for NG infection following vaccination was 0·70 (95% CI 0.38–1.27; *p* = 0.238) (Supplementary Table 1). Number of casual partners (*p* = 0.007) and taking PrEP and also having had gonorrhoea in the last 3 months prior to enrolment (0.011), showed statistically significant associations, while chlamydia infection, a past history of gonorrhoea and doxycycline use were not significant factors.

There was a significant increase in casual partners and a decrease in condom use during follow up in the vaccinated compared to the unvaccinated arm. A higher proportion of vaccinated compared to unvaccinated participants reported ≥11 partners in the last 3 months (15.9% vs 6.8%; *p* < 0.001) and <50% condom use (82.6% vs 75.8%; *p* = 0.045) (Supplementary Table 2). No differences in PrEP, doxycycline use or STIs were observed between the vaccinated and unvaccinated arm during follow up (Supplementary Table 2).

### Vaccine safety and tolerability

During the study period, 98 AEs were reported (10.9% of all study visits) (Table 4). The vaccinated arm had significantly more AEs than the unvaccinated arm, with 67 events compared to 31 (*p* < 0.0001), of which the majority (24/67) were injection site reactions. There was no difference in SAEs in both arms. No vaccine-related SAEs were reported and there were no AEs that had not been reported to the TGA previously. One death, one newly acquired HIV infection, and one episode of disseminated NG infection was reported in the unvaccinated arm and were unrelated to the study intervention.

**Table 4:** Adverse events

	Total *N* (%) of total number of study visits)	Unvaccinated (*N* = 66)	Vaccinated (*N* = 64)	*P*-value
Total Adverse Events (AE)	98/896 (10.9)	31/459 (6.8)	67/437 (15.3)	<0.0001
AEs with ≥ 5 episodes				
- injection site reactions	24 (2.7)	0	24 (5.5)	
-infections and Infestations	19 (2.1)	4 (0.9)	10 (2.3)	
-gastrointestinal disorders	12 (1.3)	4 (0.9)	8 (1.8)	
-musculoskeletal and connective tissue	7 (0.8)	3 (0.7)	4 (0.9)	
-renal and urinary disorders	7 (0.8)	5 (1.1)	2 (0.5)	
-skin and subcutaneous disorders	6 (0.7)	4 (0.9)	2 (0.5)	
Grade 3-5 Adverse events	20 (2.2)	12 (2.6)	8 (1.8)	0.427
Vaccine related AE	31 (3.5)	n/a	31 (7.1)	n/a
-administration site reactions/ pain	24 (2.7)	n/a	24 (5.5)	
-flu-like symptoms/ fever	4 (0.4)		4 (0.9)	
-nausea	2 (0.2)		2 (0.5)	
-herpes simplex reactivation	1 (0.1)		1 (0.2)	
Vaccine related Grade 3-5 AE or SAE	0	n/a	0	n/a
All Serious Adverse Events (SAE)	19 (2.1)	12 (2.6)	7 (1.6)	0.293
-SarsCoV-2 infection	2 (0.2)	-	2 (0.5)	
-genitourinary disorders	7 (0.8)	4 (0.9)	3 (0.7)	
-disseminated NG infection	1 (0.1)	-	1 (0.2)	
-new HIV infection	1 (0.1)	1 (0.2)	-	
-gastrointestinal disorders	5 (0.6)	5 (1.1)	-	
-death	1 (0.1)	1 (0.2)	-	
-other	2 (0.2)	1	1 (0.2)	

Adverse event grouped by the Medical Dictionary for Regulatory Activities (MedRA) Primary System Organ Class (SOC) as per Common Terminology Criteria for Adverse Events (CTCAE) v5.0.

### Vaccine immunogenicity against NG

**Fig. 3:**
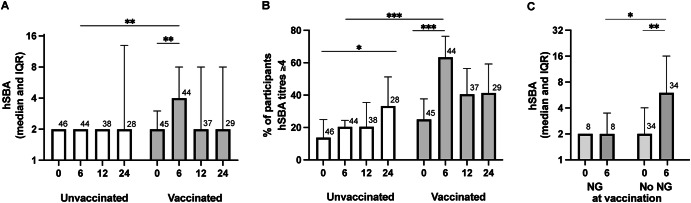
Human serum bactericidal activity (hSBA) of participant sera. **A** hSBA titres (median and interquartile range (IQR)) and **B** proportion of participants with hSBA titre ≥4 at each study visit. **C** hSBA titres in vaccinated participants with *Neisseria gonorrhoeae* (NG) infection or with no NG infection at the time of vaccination. *p < 0.05; **p < 0.01: ***p < 0.001. Numbers above the bars indicate sample size.

The median hSBA titre was significantly higher in the vaccinated arm compared to the unvaccinated arm at 6 months (4 [IQR 2–8[ vs 2 [IQR 2–2]; *p* = 0.0021) (Fig. 3A). There was also a significant increase in median hSBA titres from baseline to 6 months in the vaccinated group (2 [IQR 2–3] to 4 [IQR 2–8]; *p* = 0.0007), but not in the unvaccinated arm (2 [IQR 2–2 to 2 [IQR 2–2]; *p* = 0.354) (Fig. 3A). A significantly higher proportion of participants had hSBA titres ≥4 in the vaccinated arm compared to the unvaccinated arm at 6 months (63.4% vs 20.5%; *p* < 0.001) (Fig. 3B).

In the vaccinated arm, 13/64 (20·3%) participants receiving their first 4CMenB dose and 3/60 (5.0%) participants receiving their second dose had NG infection at the time of vaccination. For the 42 vaccinated participants with hSBA data available for both baseline and 6-month visits (3 months post dose 2), the median hSBA titre was significantly higher at 6 months in those who were negative for NG infection (6 [IQR 2-16] vs 2 IQR 2–4]; *p* = 0.0355). There was also a significant increase in titres from baseline to 6 months in those who were negative for NG infection (2 [IQR 2–4] to 6 [IQR 2–16]; *p* = 0.0024), but not those with NG infection at the time of receiving the first or second vaccine dose (2 [IQR 2–2] to 2 [IQR 2–4]; *p* > 0.999)(Fig. 3C). The proportion of participants with an increased hSBA titre at 6 months relative to baseline was 52.9% (18/34) among those with no NG infection at the time of vaccination, compared to 14.3% (1/7) among those with NG infection (*p* = 0.099). There was no significant difference in hSBA titres at 6 months for participants with NG in the 3 months before vaccination or with a history of NG compared to those with no NG before or at the time of vaccination (*p* > 0·999)

## Discussion

In the MenGO study, 4CMenB vaccination did not significantly reduce the incidence of gonorrhoea, with rates of 34.9 cases per 100 PY in the unvaccinated arm and 27.1 cases per 100 PY in the vaccinated arm (IRR 0.78 [95% CI 0·40–1·51]; *p* = 0.457; aIRR 0.66 [95% CI 0.36–1.23]; *p* = 0.191). This finding is consistent with the DOXYVAC trial (adjusted hazard ratio of 0·78 [95% CI 0.60–1.01; *p* = 0·061])^16^. However, neither trial was sufficiently powered to provide definitive evidence of vaccine efficacy. Our study does provide evidence that 4CMenB induces antibodies with bactericidal activity against NG. Nevertheless, these immune responses were not sustained at 24 months and were lower in individuals with an NG infection at the time of vaccination. The 4CMenB vaccine is safe, with no vaccine-related AEs not previously reported to the TGA and no vaccine-related SAEs identified during 24 months of follow-up.

The MenGO and the DOXYVAC trials^16^ both included GBM, who, because of frequent partner change and low condom use, were likely to experience frequent exposures to NG and other STIs, a high proportion of extragenital NG infection and concurrent NG/chlamydia infections, and have active NG infection at the time of first vaccination. In comparison, observational studies from mass vaccination programmes describe a younger population, with lower NG risk, and where extragenital screening for NG is not routinely recommended^11,14,15,27,28^. These are key differences that may impact vaccine efficacy in these different populations.

Our study is the first 4CMenB RCT to investigate immunological responses to NG following vaccination. As there is no established immunological correlate of protection for NG infection, these exploratory immune-response findings cannot be used to predict clinical outcomes. A hSBA titre ≥4 is, however, an accepted surrogate of protection for meningococcal disease and licensure of 4CMenB and MenACWY vaccines were based on hSBA data^29^. We show that 4CMenB induces a functional immune response against NG, particularly within the first 3 months after vaccination. Even though hSBA titres remained higher than baseline throughout the study in the vaccinated arm, they waned over time, so that there was little difference in hSBA titres between the vaccinated and unvaccinated arms at 12 and 24 months. Recent observational data support at least 5 years of protection against both meningococcal disease and gonorrhoea in adolescents, but also suggest reduced vaccine efficacy after 2 years^30^. Similar trends in waning of hSBA titres against meningococcal B were observed following 4CMenB vaccination in infants and toddlers^31^ and similarly in a modelling study of vaccinated adolescents^32^. Both arms exhibited a gradual rise in hSBA over time, which may reflect recurrent NG exposure and/or cross reactive antibodies from other antigenic stimuli (e.g., carriage of *N. meningitidis*).

The high incidence of NG infection seen in MenGO and DOXYVAC may impact vaccine efficacy in GBM. An important, previously unreported observation in our study was the impact on immunological responses of concurrent NG infection at the time of receiving the first or second dose of 4CMenB. Interestingly, GBM with an NG infection at the time of vaccination had lower median hSBA titres and a lower proportion of participants with an increase in hSBA titre at 6 months compared to baseline than those without NG infection. NG infection is known to modulate immune responses and can induce antibodies that may block SBA against NG (e.g., antibodies to reduction modifiable protein (Rmp))^33^. A similar mechanism may be responsible for the lower SBA responses to 4CMenB in individuals with acute NG infection at the time of vaccination. Whether this translates to protection against NG infection, however, remains to be established. Larger studies are needed to correlate hSBA titres with protection against NG infection and to confirm the role of NG infection at the time of vaccination (e.g., NCT04415424, NCT04722003, NCT05294588 clinicaltrials.gov.au). In the meantime, our findings have important implications for countries considering 4CMenB vaccination for GBM at high risk of gonorrhoea, similar to the UK and Galicia^19,20^. Pending further evidence from larger RCTs, our results indicate that it might be prudent to delay 4CMenB vaccination in GBM with active NG infection at least until after they have completed treatment and/or recovered from their infection.

Our study observed significant behavioural difference between groups during follow up, with a higher proportion of participants in the vaccinated arm who reported ≥11 casual partners and less condom use. This behavioural shift in the vaccinated group may have been due to the open-label study design, although a similar shift was not observed in the open-label DOXYVAC trial^16^. While behaviour changes may add uncertainty to our interpretation, they do not directly explain the outcomes observed. The impact of risk compensation and behaviour modification after vaccination has yet to be reported, but is an important consideration for determining vaccine efficacy and uptake needed to achieve population level reduction in gonorrhoea infection. Following the implementation of HIV-PrEP in Australia, for example, a compensatory reduction in condom use was observed^34^, although the incidence of bacterial STIs did not significantly increase^3^.

A limitation of our study was the relatively small sample size, limited statistical power, and its single-centre design. The COVID-19 pandemic-associated physical and social restrictions significantly impacted our study, and we did not achieve the 95% retention rate expected, with only 86.4% and 89.1% participants included in the final analysis in the unvaccinated and vaccinated arms, respectively. Attrition rates and missed study visits were similar in both arms and contributed to our study being underpowered. Although compliance with NG testing at attended study visits was very high (≥96%), up to 26% of scheduled study visits were missed, which would result in asymptomatic infections remaining undetected. Indeed, a shift in health seeking behaviour was observed during COVID-19, including accessing sexual health services based on clinical urgency (e.g., symptoms) over asymptomatic screening^35^, which might also explain the relatively high rates of symptomatic infections (49% in MenGO vs 10% in DOXYVAC). Our study also observed a relatively low rate of partner change in keeping with nationwide trends, which saw a 58% reduction in the average number of partners during COVID-19 restrictions^36^. Other considerations that are important to understand vaccine efficacy, including antimicrobial resistance and duration of infection, were not included in this study, and the population was under-represented for PLHIV and First Nation Australians who have been identified as key target group at risk of NG^2^.

Our study, which was powered to detect a vaccine efficacy of at 67% or higher, did not demonstrate a significant reduction in NG infection in GBM following 4CMenB vaccination. Exploratory immunogenicity data suggest that 4CMenB can induce an immune response against NG, but bactericidal titres waned over time and were lower in those with NG infection at the time of vaccination. However, in the absence of a known immunological correlate of protection for NG infection, this immunogenicity data cannot be used to explain clinical outcomes. These findings provide important insights for current 4CMenB programs aimed at gonorrhoea prevention in GBM and highlight the need for further research to understand the impact of past or current NG infections on vaccine-induced immunity. Further research is required to advance the development of effective gonorrhoea vaccines and must consider implementation strategies that maximise protection in high-risk populations.

## Supplementary information

Supplementary information

## Data Availability

The study protocol has already been published^21^. Raw datasets are not publicly available due to the inclusion of sensitive individual participant data and privacy restrictions. However, data requests for de-identified individual participant data, statistical analysis plan, data dictionary and statistical codes can be submitted to the corresponding authors and will require approval from the Gold Coast Health Human Research Ethics Committee. Data access and confidentiality agreements with the sponsor will be required for accessing shared data in keeping with Queensland Health and Griffith University policies.
