# Supplementary material for: Efficacy of the meningococcal vaccine against Neisseria gonorrhoeae: a randomised clinical trial (MenGO)
Source: NPJ Vaccines. 2026 Apr 22;11:162. doi: 10.1038/s41541-026-01422-y (PMC13470016; doi:10.1038/s41541-026-01422-y)
Supplement: Supplementary file 1 [file 41541_2026_1422_MOESM1_ESM.pdf]

Efficacy of the 4CMenB meningococcal vaccine against *Neisseria gonorrhoeae*: a randomised clinical trial (MenGO)

Caroline Thng[1,2*], Sharareh Eskandari[2], Fengyi Jin[3], Ian Hughes[4], Andrew E. Grulich[3], Maree O'Sullivan[1], Evgeny A. Semchenko[2] and Kate L. Seib[2*]

[1] Gold Coast Sexual Health, Southport Community Health Precinct, Southport, Qld 4215, Australia
[2] Institute for Biomedicine and Glycomics, Griffith University, Southport, Qld 4215, Australia
[3] The Kirby Institute, University of New South Wales, Sydney, New South Wales, Australia
[4] Gold Coast University Hospital, Southport, Qld 4215, Australia

*Correspondence: Caroline Thng, Gold Coast Sexual Health Service, Southport, 4215, Australia caroline.thng@health.qld.gov.au

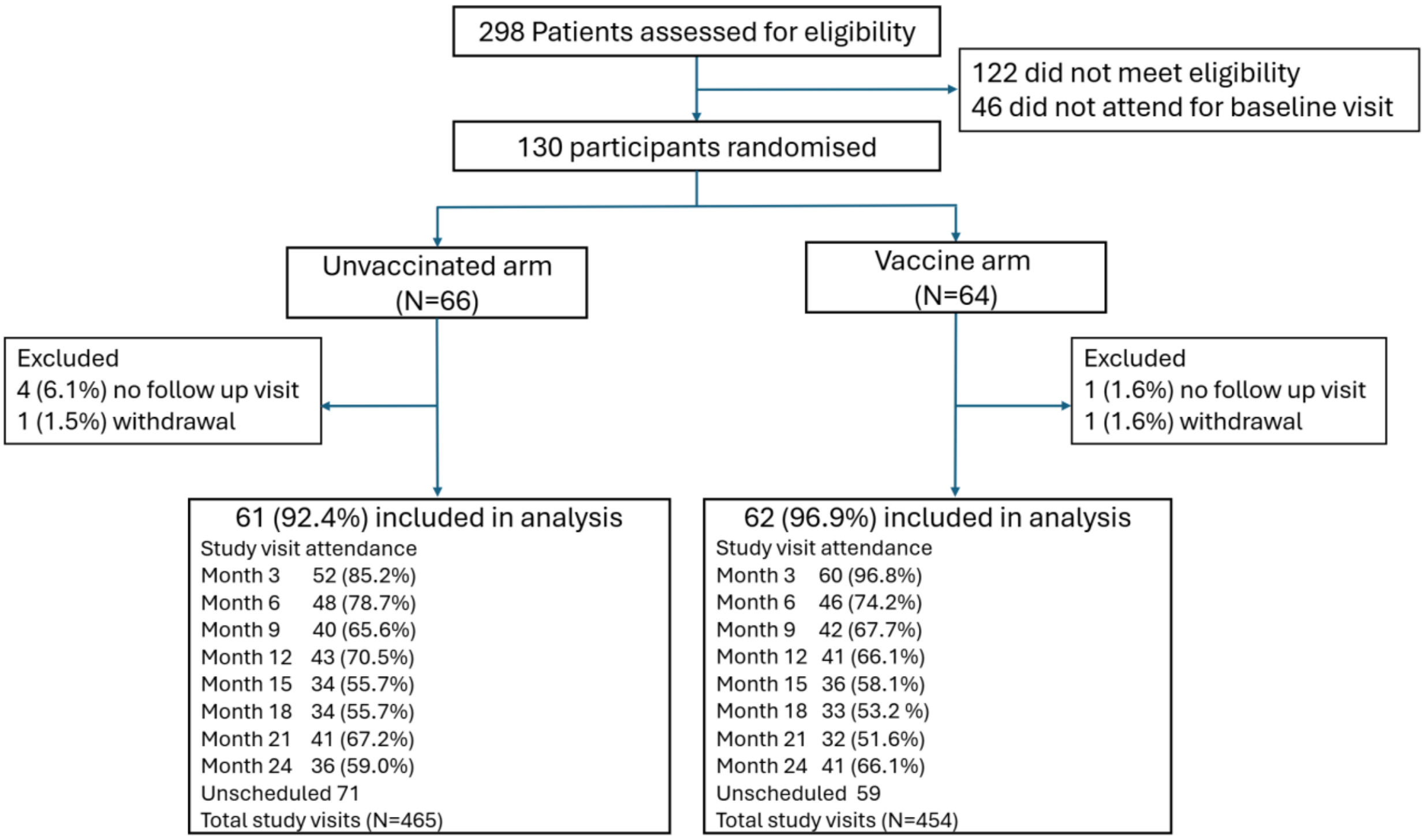

Supplementary Figure 1: Trial profile – Intention to treat analysis (ITT).
Follow up was defined any visit after baseline. Percentages in follow up describe the proportion of participants attending each study visit.

Supplementary Table 1: Multivariable analysis of vaccine efficacy and other risk factors for NG infection

	Adjusted IRR	95% CI	P value
**Arm**			0.238
Unvaccinated	1	---	
Vaccinated	0.70	0.38-1.27	
**Number of casual partners***			0.007
0-5	1	---	
6-10	1.83	0.96-3.49	
>10	2.43	1.21-4.87	
**Baseline inclusion criteria**			0.011
Taking PrEP	1	---	
Gonorrhoea ^	1.36	0.55-3.37	
Both	2.53	1.23-5.18	

* Indicates time-varying variables captured at every study visit.
^ Gonorrhoea diagnosis in the 3 months prior to enrolment.
Only variables with p-value <0·50 have been retained and presented

Supplementary Table 2: Demographic and STIs by allocation group during follow up period.

	Unvaccinated n (%)	Vaccinated n (%)	P value
**HIV Status**			0.352
Negative	54 (94.7)	55 (96.5)	
Positive	3 (5.3)	2 (3.5)	
**Casual partners (last 3 months)**			0.002
0-5	237 (76.2)	205 (67.9)	
6-10	53 (17.0)	49 (16.2)	
11 or more	21 (6.8)	48 (15.9)	
**Condom use**			0.045
<50%	226 (75.8)	237 (82.6)	
≥50%	72 (24.2)	50 (17.4)	
**Other STIs**			0.199
Chlamydia	39 (10.1)	51 (13.5)	
Syphilis	7 (1.8)	9 (2.4)	
Concurrent NG/ CT	7 (1.8)	4 (1.1)	
**PrEP use (HIV-negative only)**			0.456
No	27 (47.4)	31 (54.4)	
Yes	17 (52.6)	14 (45.6)	
**Doxycycline use**			0.975
No	332 (85.6)	324 (85.5)	
Yes	56 (14.4)	55 (14.5)	
